# Protective Effects of Caffeic Acid Phenethyl Ester on Fluoxetine-Induced Hepatotoxicity: An Experimental Study

**DOI:** 10.1155/2016/1247191

**Published:** 2016-04-10

**Authors:** Ahmet Yılmaz, Bilal Elbey, Ümit Can Yazgan, Ahmet Dönder, Necmi Arslan, Serkan Arslan, Ulaş Alabalık, Hamza Aslanhan

**Affiliations:** ^1^Department of Family Physicians, Faculty of Medicine, Dicle University, 21280 Diyarbakir, Turkey; ^2^Department of Immunology, Faculty of Medicine, Dicle University, 21280 Diyarbakir, Turkey; ^3^Department of Physiology, Faculty of Medicine, Zirve University, 27260 Gaziantep, Turkey; ^4^Department of Biochemistry, Faculty of Medicine, Dicle University, 21280 Diyarbakir, Turkey; ^5^Department of Pediatric Surgery, Faculty of Medicine, Dicle University, 21280 Diyarbakir, Turkey; ^6^Department of Pathology, Faculty of Medicine, Dicle University, 21280 Diyarbakir, Turkey

## Abstract

*Background*. The aim of the study was to analyse the effect of caffeic acid phenethyl ester (CAPE) on fluoxetine-induced hepatotoxicity in rats.* Materials and Methods*. Group I served as control. Group II received CAPE intraperitoneally. Group III received fluoxetine per orally. Group IV received fluoxetine and CAPE. The total antioxidant capacity (TAC), total oxidant status (TOS), oxidative stress index (OSI), and liver enzymes including paraoxonase-1 (PON-1), aspartate transaminase, and alanine transaminase levels were measured. Liver tissues were processed histopathologically for evaluation of liver injury and to validate the serum enzyme levels.* Results*. An increase in TOS and OSI and a decrease in TAC and PON-1 levels in serum and liver tissues of Group III were observed compared to Groups I and II. After treatment with CAPE, the level of TOS and OSI decreased while TAC and PON-1 increased in serum and liver in Group IV. Histopathological examination of the liver revealed hepatic injury after fluoxetine treatment and reduction of injury with CAPE treatment.* Conclusion*. Our results suggested that CAPE treatment provided protection against fluoxetine toxicity. Following CAPE treatment with fluoxetine-induced hepatotoxicity, TOS and OSI levels decreased, whereas PON-1 and TAC increased in the serum and liver.

## 1. Introduction

Fluoxetine is a selective serotonin reuptake inhibitor (SSRI) as a first-line drug which is used for treatment of depression and many neuropsychiatric disorders [1]. Use of such drugs for suicidal purposes due to easy availability of these drugs by the patients has been reported [2–6]. Fluoxetine is a fluorine-including SSRI drug acting as an antidepressant agent with high absorption after oral administration; this agent is metabolized in the liver and excreted by the urine [7]. It is a safe and well-tolerated drug; however, adverse events are observed with high doses. Such adverse events include gastrointestinal problems (nausea, vomiting, and diarrhea); nervous system problems (insomnia, anxiety), and sexual dysfunction, bipolar disorders, balance disorder, metabolic disorders (hyponatremia), and organ failure (liver failure, renal failure) in rare cases [6, 8]. Studies carried out on rats reported hepatic ischemia and necrosis in the study groups in which fluoxetine was administrated in 7.5 mg/kg, 10 mg/kg, and 25 mg/kg doses, respectively [9]. Hepatocellular hydropic degeneration, hepatomegaly, steatosis, lobular inflammation, focal necrosis, cholestasis, portal zone inflammation, and increase in oxidative stress were reported with aforesaid doses of fluoxetine [9–11]. Some traditional drugs which have pleiotropic biological activity are currently used as supplements for treatment of different diseases such as cholestasis, hepatosteatosis, and hepatic ischemia [12, 13].

The studies on caffeic acid phenethyl ester (CAPE) have demonstrated that propolis, which is the active component in CAPE and used in traditional medicine, has antiviral, anti-inflammatory, immunomodulatory, and antioxidant characteristics. Also, CAPE has inhibitor effects on lipid peroxidation, as well as lipoxygenase and cyclooxygenase enzymes [14–16]. CAPE is a flavonoid group compound and use of CAPE in traditional medicine for a long period acts as a bioactive component of the propolis in the hives [11]. CAPE includes two cyclic structures, one of which has two hydroxyl groups [17]. Such hydroxyl groups actively act in redox reactions and create the antioxidant characteristics [18].

Total oxidant status (TOS), total antioxidant capacity (TAC), paraoxonase-1 (PON-1), and OSI (Oxidative Stress Index) are usually measured to determine the toxicity level in damaged tissues [19]. Local hemorrhage and necrosis, neutrophil infiltration, congestion, sinusoidal extension, and local hepatocellular vacuolization which may be shown by histopathological examination indicate fluoxetine-induced hepatotoxicity [9].

The objective of the present study was to analyse the effects of CAPE as an antioxidant against the hepatotoxicity caused by fluoxetine through analysis of TOS, TAC, PON-1, OSI, AST, and ALT and histopathological examination.

## 2. Material and Method

### 2.1. Drugs

Fluoxetine hydrochloride with code number PHR1394 and CAPE with catalog number C8221 were obtained from Sigma-Aldrich Co., LLC, Germany. Fluoxetine was diluted by saline solution. Dimethyl sulfoxide (DMSO) was used as a solvent for CAPE and the solution was diluted with saline.

### 2.2. Animals and Treatment

The authors acknowledge that the aforesaid experiments on animals were carried out in accordance with applicable laws and legislations of the Republic of Turkey. The study protocols were investigated and approved by the Ethics Committee on Animals of the University (approval number 2013/49; decision number 4; date, 10.10.2013). Four groups including 8 rats which were monitored in individual cages at a room temperature of 22 ± 2°C were designed. Male Sprague Dawley rats (with a weight ranging between 320 and 340 g) used in the present study were kept under standard laboratory conditions and fed by standard rat feed and water.

The procedure started with narcotization of the rats through intramuscular injection of ketamine hydrochloride on a dose of 80 mg/kg (Ketalar, Parke Davis, Eczacibasi, Istanbul, Turkey). Skins of the rats were shaved and 10% povidone iodine solution (Betadine) was used for sterilization.

### 2.3. Rat Study Groups

The present study was conducted in rats divided into four groups of 8 animals in each group. Group I served as control and received no drug or agent. Group II received CAPE intraperitoneally at 10 mmol/kg/day for 7 days. Group III received fluoxetine 10 mg/kg/day for 7 days. Group IV received fluoxetine 10 mg/kg/day plus CAPE 10 mmol/kg/day for 7 days. The total antioxidant capacity (TAC), total oxidant status (TOS), Oxidative Stress Index (OSI), and liver enzymes including paraoxonase-1 (PON-1), aspartate transaminase (AST), and alanine transaminase (ALT) were measured. Liver tissues were processed histopathologically for evaluation of liver injury and to validate the serum enzyme levels.

### 2.4. Collection of Serum and Tissue Samples

Following final application of the medication at day 8, blood samples were collected directly from the heart through cardiac puncture. The blood samples were centrifuged at 4000 rpm for 10 minutes. Measurement of ALT and AST activities in the serum was performed according to the Reitman-Frankel method for all groups [20, 21]. Enzyme activity is expressed in International Units per liter (IU/L).

Tissue samples were obtained from the liver for histopathological examination. The tissues were prepared for biochemical analysis by clearing the residues and blood with saline solution. Additionally, the tissue samples were stored in plastic containers with 10% formaldehyde solution for histopathological examination. The tissue specimens were stored at −80°C, and transfer of samples was performed in dry ice. The specimens with an approximate weight of 0.30 to 0.50 g were placed into a tube to which 2 mL of Tris-HCl buffer was added. The tubes were placed in an ice-filled plastic container, and they were treated with a 50 mM phosphate-buffered saline with a pH of 7.0 in a homogenizer at 14,000 rpm for 1 to 3 minutes (Hg-15 A Model Analog Homogenizer, Wisd Laboratory/Instruments, Daihan Scientific Co., Ltd., Korea). The homogenate was centrifuged for 30 min. at 15,000 rpm, at +4°C. Supernatant samples were used for analysis of TAC, TOS, and PON-1.

### 2.5. Analysis of TAC and TOS

TAC and TOS values were detected through automated biochemical measurement using a novel measurement method developed by Erel [22]. Methods like measuring TAC are generally calibrated using a standard antioxidant solution called Trolox Equivalent. TAC measurements were performed by kinetic reading in the spectrophotometer 5 minutes after the sample and reagent were mixed. Measured TAC values were read as mmol Trolox Equiv per liter for serum and mmol Trolox Equiv per gram of protein for tissue samples. TOS was measured by reading at end-point 560 nm in the spectrophotometer after 3-4 minutes following mixing the samples and reagents, and the results were expressed as amount of hydrogen peroxide per liter (*μ*mol H_2_O_2_ equiv/L) for serum and hydrogen peroxide per gram (*μ*mol H_2_O_2_ equiv/gram) of protein for tissue samples.

### 2.6. Paraoxonase Activity

Paraoxonase activity of PON-1 was measured with a commercial kit of Rel Assay Diagnostics (Gaziantep, Turkey) using a spectrophotometric method. The kit contained both substrate solution and Tris buffer. The substrate solution consisted of paraoxon and buffer solution [23]. Activity was determined according to the kit instructions. The linear increase of* p*-nitrophenol at 412 nm wavelength absorbency which was revealed by hydrolysis of paraoxon in the sample was accepted as the PON-1 activity [24]. The molar absorptivity of* p*-nitrophenol is 18,290 nm and one unit of paraoxonase activity equals 1 mol of paraoxon hydrolyzed per liter per minute at 37°C. U/L for serum and U/gr protein for tissue samples were used as a unit of PON-1 activity.

### 2.7. Calculation of OSI

Following the TAC and TOS measurements, the OSI levels were calculated by means of the following formula: OSI = (TOS)/(TAC). Oxidative Stress Index (OSI) is an indicator parameter of the degree of oxidative stress [25].

### 2.8. Biochemical Analyses

Total antioxidant capacity (TAC), total oxidant status (TOS), paraoxanase-1 (PON-1), Oxidative Stress Index (OSI), ALT, and AST were analysed in the blood sample. Histopathological analysis as well as biochemical parameters, TAC, TOS, and PON-1, and OSI levels to evaluate oxidative stress were performed on the liver tissue samples [20, 21].

### 2.9. Histopathological Examination

Tissues were carried in 10% formalin, and the paraffin blocks were prepared in 4 *μ*m slices. Tissues were stained with hematoxylin and eosin using standard protocol. The sections were examined under a light microscope using 200x magnification for assessment of the degree of liver injury by a liver pathologist blinded to the animal grouping.

*Liver Injury Score*. Liver injury scores were as follows.

 Grade 0: none or slight injury,
 Grade 1: slight injury; cytoplasmic vacuolization and nuclear pyknosis,
 Grade 2: moderately increased nuclear pyknosis, increased loss of eosinophil, and intracellular margin in the cytoplasm,
 Grade 3: severe injury, hemorrhage, neutrophil infiltration, and severe necrosis causing disintegration of liver cells.

The tissue injury through the grading above following histopathological analysis [26] was detected and analysed statistically.

### 2.10. Statistical Analysis

Statistical analysis was performed using SPSS for Windows 11.5 (SPSS Inc., Chicago, IL, USA). Data were presented with descriptive statistics including mean ± standard deviation (SD) for parametric data and median (range between 25% and 75%) for nonparametric data. The normality of the parameters in serum and tissue groups was analysed with the Shapiro-Wilk test. The parametric data were analysed with an Anova test and a Tukey post hoc test for binary comparisons. The nonparametric data were analysed with a Kruskal-Wallis test, and a Mann-Whitney *U* test was used for binary comparisons. A *p* value of <0.05 was accepted as statistically significant.

## 3. Results 

**Figure 1:**
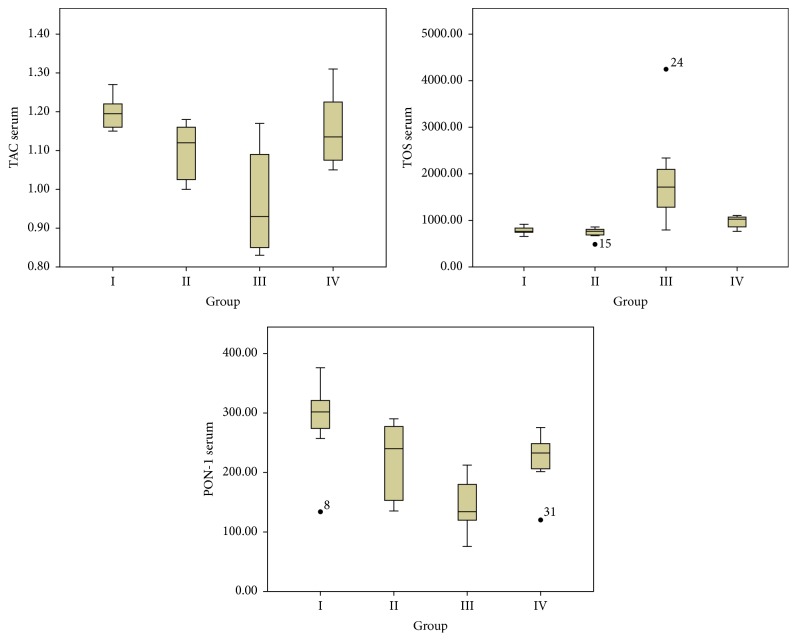
The biochemical parameters obtained from serum samples. Total antioxidant capacity (TAC), total oxidant status (TOS), and paraoxonase-1 (PON-1).

**Figure 2:**
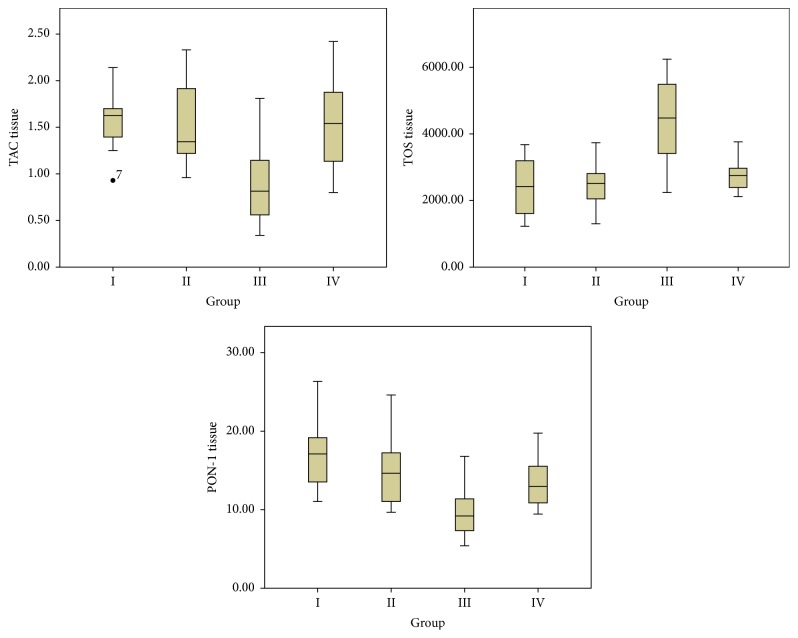
The biochemical parameters obtained from liver tissue samples. Total antioxidant capacity (TAC), total oxidant status (TOS), and paraoxonase-1 (PON-1).

**Figure 3:**
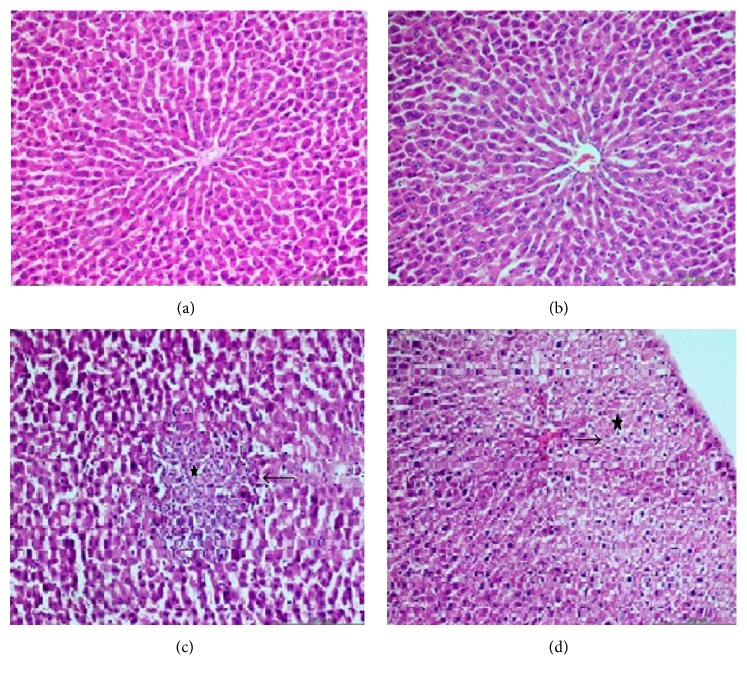
Histopathological changes in liver tissue. (a) Normal liver tissue (H&E stain, 200) was shown in sham group. (b) Normal liver tissue (H&E stain, 200) was shown in control group. (c) Slight histological changes (arrow) and increased nuclear pyknosis (stars) were shown in Group III (H&E stain, 200). (d) Liver tissue was normal other than mild sinusoidal dilatation (star), and nuclear vacuolization (arrow) in Group IV (H&E stain, 200).

The biochemical parameters obtained from serum samples and statistical evaluations between the groups are presented in Table 1. The decrease in TAC and PON-1 levels, as well as the increase in TOS, OSI, and histopathological scores in Group III when compared with Groups I and II, was statistically significant (*p* < 0.05). The decrease in TOS, OSI, ALT, and AST levels and the increase in TAC and PON-1 levels were observed in Group IV when compared with Group III (*p* < 0.05) (Table 1 and Figure 1). The biochemical parameters obtained from liver tissue samples and statistical evaluations between the groups are presented in Table 1 and Figure 3. A statistically significant decrease in TAC and PON-1 levels as well as an increase in TOS, OSI, and histopathological scores was observed in Group III when compared with Groups I and II (*p* < 0.05) (Table 1 and Figure 2). After the administration of CAPE, the oxidative stress was significantly ameliorated, TOS and OSI were decreased, and TAC and PON-1 were increased in the liver tissue. The histopathological examination revealed that liver tissue injury after fluoxetine administration decreased after administration of CAPE; however, such a finding was not statistically significant (Table 1 and Figure 2). The histopathological examination revealed that liver slices of the rats have a normal histological appearance in the sham (Figure 3(a)) and control groups (Figure 3(b)). Slices of the rats in Group III presented modifications including slight histological disruption; slight injury; and moderate injury by cytoplasmic vacuolization, increased nuclear pyknosis, increased eosinophil, and intracellular margin loss (Figure 3(c)). Although the modifications observed in Group IV (Figure 3(d)) were less than those for Group III, they were not statistically significant.

## 4. Discussion

Our results demonstrate that CAPE treatment decreased liver injury and serum oxidant enzyme levels caused by fluoxetine treatment. CAPE treatment also increased antioxidant enzyme levels in the present study. Furthermore, positive contribution of CAPE to recovery of hepatic modifications caused by fluoxetine was observed at tissue level. This indicated the benefit of CAPE on liver injury induced by fluoxetine.

Increase of transaminase enzyme activity (AST, ALT) in the serum as a sensitive indicator for liver injury was reported in hepatic cell injury [27]. Several studies reported an increase in serum transaminase levels after a high dose of fluoxetine exposure [10, 28]. Higher serum transaminase levels were detected due to fluoxetine. A decrease in serum transaminase levels was observed with CAPE treatment in the present study. Such observations show the hepatotoxicity reducing effect of CAPE.

Fluoxetine includes fluorine; if a drug has fluorine in its compound, increase in pharmacokinetic and pharmacodynamic characteristics along with toxic effects appears [9]. One of the side effects of long-term fluorine drugs is liver injury [29, 30]. Fluoxetine is metabolized in the liver by cytochrome p450 and converted into norfluoxetine and several metabolites [31]. Dose-dependent increase of liver injury was reported with fluoxetine [9]. Souza et al. showed in their study that high-dose fluoxetine has toxic effects [32]. Inkielewicz-Stêpniak, investigating the dose-dependent effect of fluoxetine on the liver, reported a dose-dependent increase of liver injury in the groups by administrating 8 mg/kg and 24 mg/kg of fluoxetine [10]. Experimental studies indicated that fluoxetine decreased antioxidant levels, increased oxidant stress levels by elevating superoxide anion levels, and induced oxidative stress [33–35]. In the present study, we detected modifications varying from slight histological disruption in liver cells to mononuclear cell infiltration, hemorrhage, degeneration in fat cells, and apoptotic changes progressing to necrosis following administration of fluoxetine in toxic doses.

OSI measurements provide a sensitive, novel index of oxidative stress and can reflect both oxidative and antioxidative parameters [36]. In the present study, OSI was lower in the fluoxetine plus CAPE group than in the fluoxetine only group.

PON-1 is an antioxidant enzyme that prevents oxidation of low-density lipoprotein. Activity of PON-1 decreases with an increase of oxidative stress [37, 38]. Uzar et al. showed in their study that CAPE prevented a decrease in PON-1 levels in experimental animals which developed neurotoxicity; furthermore, recovery was observed in oxidative stress parameters and histopathological examination of the injured tissues [39]. In the present study, high doses of fluoxetine administration reduced PON-1 level; a reincrease of PON-1 levels was observed with administration of CAPE as a protective agent against fluoxetine. The present study suggests that CAPE provides significant prevention of PON-1 activity by dissolving the free radical agents which are produced by fluoxetine.

Studies on CAPE, the active component of propolis used in traditional medicine, showed antiviral, anti-inflammatory, immunomodulatory, and antioxidant characteristics of CAPE due to inhibitor effects on lipid peroxidation as well as lipoxygenase, cyclooxygenase enzymes [14–16]. Çakir et al. reported a decrease in toxic effects on the liver due to lipid peroxidation induced by methotrexate and neutrophil infiltration caused by oxidation in the hepatic cells through the decreasing effect of CAPE [15]. Although there is not any statistically significant difference on the hepatic effects of fluoxetine, liver injury scores were lower in the CAPE-administrated group in the present study. Biochemical and histopathological findings of the present study suggested that CAPE is protective against the toxic effects of fluoxetine in the liver.

Johnston and Wheeler reported a case of serious chronic hepatitis due to fluoxetine exposure [40], whereas Cai et al. documented two cases with acute hepatitis as a result of fluoxetine therapy [41] and Cosme et al. presented a case with fluoxetine-induced acute cholestasis and confirmed such cases with a liver biopsy [42]. Furthermore, Özden et al. showed oxidative modifications histopathologically on the liver caused by fluoxetine administrated in varying doses [9]. In the present study, we detected modifications varying from slight histological disruption in liver cells to mononuclear cell infiltration, hemorrhage, degeneration in the fat cells, and apoptotic changes progressing to necrosis following fluoxetine. There was a decrease on edema and vascular congestion which appeared because of fluoxetine following treatment with CAPE; however, such findings were not statistically significant.

## 5. Conclusion

The present study indicates an increase in serum TOS, OSI, PON-1, and transaminase activity along with a decrease in TAC levels following hepatotoxicity caused by fluoxetine. Furthermore, moderate injury appeared histopathologically. CAPE was effective at reducing serum transaminase levels and oxidative stress parameters, at increasing antioxidant stress parameters, and at reversing the histopathological injury. The data presented above concludes that CAPE acts as an agent to protect the liver against oxidative alterations due to hepatotoxicity. We believe that CAPE may be used for fluoxetine-induced hepatotoxicity and that further clinical and laboratory surveys are required.

## Figures and Tables

**Table 1:** Oxidative and antioxidative parameters in rats according to the groups.

		Group I Mean ± SD	Group II Mean ± SD	Group III Mean ± SD	Group IV Mean ± SD	*p* ^**∗**^
Serum	PON-1	287.99 ± 70.87	220.71 ± 65.18^a^	144.41 ± 47.05^aa,b^	221.27 ± 46.97^a,c^	0.002
	TAC	1.19 ± 0.041	1.09 ± 0.07^a^	0.96 ± 0.1^aa^	1.15 ± 0.09^c^	0.003
	TOS	782.62 ± 79.85	732.95 ± 116.33	1903.35 ± 1053^aa,bb^	973.2 ± 126.58^aa,bb,cc^	0.0001
	AST	96.87 ± 11.38	109 ± 37.39	166.12 ± 45.83^aa,b^	98.50 ± 21.53^cc^	0.003
	ALT	57 ± 7.11	74.62 ± 16.93^a^	119 ± 51.95^aa,b^	76.37 ± 20.33^cc^	0.002
	OSI	655 ± 77.3	672.15 ± 128	2039.51 ± 1226^aa,bb^	853.45 ± 158.26^a,cc^	0.001

Tissue	PON-1	17.12 ± 4.92	15.02 ± 4.89	9.75 ± 3.55^aa,b^	13.49 ± 3.35^c^	0.014
	TAC	1.56 ± 0.35	1.53 ± 0.48	0.89 ± 0.48^a,b^	1.54 ± 0.53^c^	0.041
	TOS	2418.29 ± 919.58	2473.71 ± 728.89	4406.88 ± 1342.29^a,bb^	2762.29 ± 509.23^c^	0.014
	OSI	1599.28 ± 688.02	1700.4 ± 604.73	6723.49 ± 5088.54^aa,bb^	1964.64 ± 602.77^cc^	0.002

		Median (IQR 25%–75%)	Median (IQR 25%–75%)	Median (IQR 25%–75%)	Median (IQR 25%–75%)	

	Hysto. Score	0	0	1.375 ± 0.51^d^	0.875 ± 0.64^aa,bb^	0.0001

^*∗*^Kruskal-Wallis test. Groups are as follows: Group I: control, Group II: sham (CAPE), Group III: fluoxetine 10 mg/kg, Group IV: fluoxetine 10 mg/kg + CAPE, PON-1 (U/L): paraoxonase-1, TAC (mmol Trolox Equiv/L): total antioxidant capacity, TOS (*μ*mol H_2_O_2_ equiv/L): total oxidant status, OSI (Arbitrary Unit): oxidative stress index, AST: aspartate aminotransferase, ALT: alanine transaminase (enzyme activity is expressed in International Units per liter (IU/L) for serum). PON-1 (U/gr protein), TAC (mmol Trolox Equiv/gram protein): total antioxidant capacity, TOS (*µ*mol H_2_O_2_ equiv/gram) total oxidant status, OSI (Arbitrary Unit): Oxidative Stress Index, AST: aspartate aminotransferase, ALT: alanine transaminase (enzyme activity is expressed in International Units per liter (IU/L)), SD: standard deviation for tissue. Hysto. Score: histopathologic score, and IQR: interquartile range.

^a^ *p* < 0.05 versus group I.

^aa^ *p* < 0.01 versus group I.

^b^ *p* < 0.05 versus group II.

^bb^ *p* < 0.01 versus group II.

^c^ *p* < 0.05 versus group III.

^cc^ *p* < 0.01 versus group III.

^d^ *p* < 0.001 versus groups I and II.
